# Effects of different exercise types on balance function in healthy older adults and Parkinson’s patients: a systematic review

**DOI:** 10.3389/fnagi.2024.1411584

**Published:** 2024-12-24

**Authors:** Xu Bin Guo, Lu Tang

**Affiliations:** ^1^Department of Physical Education, Civil Aviation Flight University of China, Guanghan, China; ^2^Institute of Aviation Sports, Civil Aviation Flight University of China, Guanghan, China; ^3^Integrated Sports Medicine Innovation Hub for Pilots, Civil Aviation Flight University of China, Guanghan, China

**Keywords:** Parkinson’s disease, healthy older adults, Tai Chi, yoga, resistance exercise, balance function, exercise intervention

## Abstract

**Objective:**

This study aims to compare the effects of Tai Chi, yoga, and resistance training on balance function in healthy elderly individuals and patients with Parkinson’s disease (PD). Given the well-documented benefits of these three exercise types in enhancing balance and motor function, it is crucial to assess their differential impacts.

**Methods:**

A comprehensive search was conducted across PubMed, Web of Science, Scopus, and Cochrane Library databases through December 2023. Articles were selected based on predefined criteria, screened, and evaluated by two independent researchers who also extracted study characteristics. The risk of bias was assessed using the Cochrane risk of bias tool. The primary outcome measures were the Berg Balance Scale and the Timed Up and Go test, while the secondary measure was the Unified Parkinson’s Disease Rating Scale Part III. A random effects model was employed, and heterogeneity was measured using the *I*^2^ statistic.

**Results:**

Of the 21 studies reviewed, 9 focused on healthy older adults and 12 targeted individuals with PD. The meta-analysis showed that Tai Chi, yoga, and resistance training significantly improved balance in patients with PD compared to control groups (*p* < 0.00001). Resistance training was most effective in enhancing dynamic balance in healthy older adults (*p* = 0.003), while Tai Chi had the most significant impact on balance improvement in PD patients (*p* < 0.00001). Notably, interventions conducted three to four times per week, each lasting 50–60 min and continued over 12 weeks, yielded the most substantial balance improvements.

**Conclusion:**

Comparative analyses demonstrate that Tai Chi, yoga, and resistance training significantly enhance balance and motor function. Specifically, resistance training markedly improves dynamic balance in healthy elderly individuals, while Tai Chi shows pronounced improvements in motor function and balance for PD patients. Optimal balance improvements are achieved by performing interventions three to four times per week, with each session lasting 50–60 min, over 12 weeks.

**Systematic review registration:**

https://inplasy.com/, identifier INPLASY202470042.

## 1 Introduction

As the elderly population grows, so does the incidence of Parkinson’s disease (PD), a prevalent neurodegenerative condition affecting over 1% of individuals aged 55 years and above globally (Yin et al., 2023). PD originates from the depletion of dopamine neurons within the substantia nigra. As the disease progresses, its pathology extends to neocortical and cortical areas (Parga et al., 2021), impairing cognitive and behavioral functions. Common symptoms include bradykinesia, muscle rigidity, and rest tremor (Perlmutter and Ushe, 2020), resulting in lower limb motor dysfunction, such as diminished balance, gait, and mobility (Mak and Wong-Yu, 2019). Additionally, nearly all patients experience non-motor symptoms alongside basic functional deficits (Kadastik-Eerme et al., 2015). Reports indicate that approximately 70% of PD patients are prone to falls due to reduced motor function and mobility, which may lead to severe consequences like head trauma and fractures (Bloem et al., 2001; Perez-Lloret et al., 2014). Age-related alterations in brain structure and vestibular function, compounded by PD, also affect cognition, walking, and body balance (Xie et al., 2019).

The gradual loss of dopamine neurons in the brain leads to these symptoms. As the disease progresses, motor function deteriorates and can be categorized into distinct stages: Stage 1: mild symptoms appear, typically affecting only one side of the body. Patients may experience slight tremors or bradykinesia. Stage 2: symptoms become more pronounced, involving both sides of the body, but balance remains relatively stable. Stage 3: symptoms further intensify, leading to noticeable balance issues and an increased risk of falls. Stages 4 and 5: motor function is severely compromised, requiring assistance from others for walking (Xiao and Tan, 2023; Debû et al., 2018). The motor symptoms of PD, along with their impact on balance, are complex and multifaceted. As the disease advances, it significantly affects patients’ daily lives and mental health.

The prevalence of PD is projected to exceed 1 million by 2030 due to an aging population (Marras et al., 2018), highlighting the need to optimize the care and management of PD patients. Currently, two treatment approaches exist for PD: conventional and surgical. Pharmacologic interventions involve dopamine replacement therapy or dopamine agonists, Pharmacological interventions, particularly dopamine replacement therapy and dopamine agonists, are essential in managing PD. The most commonly used medications include: 1. Levodopa: as the most widely prescribed drug for PD, levodopa effectively alleviates symptoms by replenishing dopamine levels in the brain. 2. Dopamine agonists: medications such as bromocriptine and ropinirole directly stimulate dopamine receptors. These drugs can be administered in the early stages of the disease or combined with levodopa to enhance therapeutic outcomes.

In recent years, non-pharmacological treatments have gained increasing recognition as valuable adjuncts to the management of PD. Key non-drug interventions include: Physical therapy: this approach helps improve motor function, increase muscle strength, and reduce the risk of falls. Personalized exercise programs can enhance patients’ coordination and flexibility (Witt et al., 2017; Mantovani et al., 2023). Exercise therapy: regular physical activity can improve motor symptoms and help patients maintain their ability to perform daily activities. Common exercises include gait training, balance exercises, and endurance training, in addition to surgical treatments (Obeso et al., 2010; Micó-Amigo et al., 2017). While these treatments offer partial relief from the motor symptoms of the disease (e.g., tremor, bradykinesia, and rigidity), their prolonged use is associated with side effects such as cardiovascular issues and motor instability. Moreover, patients continue to experience a decline in motor function and mobility (Manson et al., 2012; Ashoori et al., 2015; Kim et al., 2019).

The treatment of PD requires a comprehensive approach that integrates both pharmacological and non-pharmacological interventions. By tailoring treatment plans to the needs of individual patients, it is possible to manage symptoms effectively while also enhancing their quality of life (Illman, 2023). As research progresses, the effectiveness of non-pharmacological interventions is increasingly recognized, providing a broader spectrum of treatment options for patients with PD. Exercise therapy, in particular, has demonstrated significant benefits across multiple domains, including physical health, mental wellbeing, and overall quality of life (Gubert and Hannan, 2021). Physical activity is a crucial non-pharmacological intervention with a wide range of benefits. Specifically, aerobic exercise and strength training have been shown to improve muscle strength and endurance, thereby enabling individuals to perform daily activities more effectively (The Lancet, 2016). Furthermore, exercise can stimulate the production of nerve growth factors, which enhance neural connections.

Physical activity represents a crucial non-pharmacologic intervention, specifically exercise therapy, aimed at ameliorating these symptoms in PD patients. For instance, progressive resistance training (PRT) can engage both the mind and body through physical and respiratory exercises, facilitating improvements in motor function among participants (Yu et al., 2013; Vieira and Cavalcante, 2021). Similarly, Tai Chi has shown positive effects, as reported by Hackney and Earhart (2008) and Bubela et al. (2019). Yoga also contributes to these benefits, with findings from Myers et al. (2020) and Elangovan et al. (2020) supporting its effectiveness including gait, balance, functional ability, and flexibility. Tai Chi (Xiao, 2014; Hosseini et al., 2018) and Iyengar Yoga (Tiedemann et al., 2013; Elangovan et al., 2020) have proven effective in enhancing balance, refining body control, and improving daily life quality and functionality in older adults with PD (Lan et al., 2013). Additionally, these practices have demonstrated modest benefits in mitigating mild brain damage (Betker et al., 2007) and mild cognitive impairment (Hemmeter and Ngamsri, 2022).

Various forms of exercise, including resistance training, Tai Chi (Leitão et al., 2022), skiing (Müller et al., 2011; De Vries et al., 2018), and dance (Santos et al., 2020), exert positive effects on older adults and individuals with PD to varying degrees. Long-term exercise regimens significantly contribute to enhancing balance function across three distinct levels: mechanistic, functional, and behavioral. Mechanistically, several studies have demonstrated that exercise training reduces inflammatory cytokines, boosts anti-inflammatory cytokines, and stimulates the production of BDNF and NAA, thereby fostering neural plasticity and functional connectivity within the brain (Tian et al., 2017; Èekanauskaitë et al., 2020). Additionally, research indicates that exercise interventions lead to improved functional connectivity in the frontal lobe (Liu et al., 2020). Functionally, prolonged Tai Chi practice notably enhances brain network function, proprioception, and balance capabilities, while also augmenting neurotransmitter metabolism and conduction, thereby impacting cognition, motor control, and balance (Li and Harmer, 2015). Additional studies have also shown significant changes in energy conduction and neurotransmitter levels, as well as improved physical functioning in PD patients after exercise intervention (Li et al., 2022).

At the behavioral level, participants’ gait, balance, and mobility were significantly improved compared to the control group, and their quality of life was substantially improved. The same study also found that participants’ balance was significantly improved after training (Zhu et al., 2023). Different types of exercise movements have certain effects on different limb parts. On the one hand, exercise can enhance body posture control, proprioception, and visual function. Movement involves the cerebral cortex and subcortical structures, such as the cerebellum, basal ganglia, and brainstem (Takakusaki, 2017; Chen et al., 2023). On the other hand, the process of body posture control requires the synergistic involvement of multiple brain regions (Stuart et al., 2018). The cerebral cortex can modulate the excitability of subcortical structures, spinal columns, and muscles according to the body’s needs to maintain balance and postural stability (Solis-Escalante et al., 2019). Both of these mechanisms are important for improving balance function. Therefore, three different forms of exercise—Tai Chi, yoga, and resistance exercise—may affect the spinal cord, brain functional connectivity, and cortical structures by influencing body posture and breathing rhythm, among other things, to further improve participants’ mobility and motor symptoms. Therefore, the benefits of exercise for the body are profound and comprehensive.

In recent years, there has been a surge in studies investigating the impact of exercise on balance function among older adults and individuals with PD. Prior research (Xiao, 2014; Winser et al., 2018; Leitão et al., 2022) has indicated that Tai Chi, yoga, and resistance exercise can effectively enhance balance function in older adults. These studies underscore the improvement in motor function and balance across various exercise modalities. However, existing literature predominantly focuses on the effects of singular exercise types, lacking comparative analyses between different modalities. Certain reviews (ALJawaee et al., 2021; Hołaj and Chalimoniuk, 2022; Chen et al., 2023; Yu et al., 2021) concentrate solely on specific exercise forms, such as wobble board training, dance, or Tai Chi, without contrasting differences between various exercise types.

Furthermore, current studies have not elucidated the disparities in balance outcomes between healthy older adults and Parkinson’s patients following exercise interventions. Hence, the objective of this review was to compare the effects of Tai Chi, yoga, and resistance exercise on balance function between healthy older adults and individuals with PD. To achieve this, we conducted a systematic review and meta-analysis of relevant studies that evaluated these exercise modalities. By synthesizing and analyzing the data, we aimed to assess the relative effectiveness of each exercise type in improving balance and preventing falls. This analysis aimed to identify the most suitable exercise modality for enhancing balance and averting falls among healthy older adults.

## 2 Materials and methods

### 2.1 Search strategy

In this study, the reviewer (XG) conducted searches across PubMed, Web of Science, Scopus, and the Cochrane Library databases using keywords, synonyms, and a search strategy employing Boolean logic operators. The search covered literature from inception to December 2023, including four distinct fields with separate search terms (detailed in Table 1). The first search field focused on participants categorized by targeted population criteria, including terms such as “Older adult,” “Older elderly,” “Older,” “Parkinson Disease,” “Primary parkinsonism,” “Parkinsonism,” and “Idiopathic parkinsonism.” The second search field included potential synonyms for various forms of exercise, such as “Tai Ji” (including “Tai chi,” “Chi,” “Tai,” “Tai Ji Quan,” “Tai Chi Chuan,” “Ji Quan,” and “Tai”), “Yoga,” and “Resistance training” (including “Resistance exercise” and “Strength training”). The third search included synonyms related to the balance function, such as “Balance” and “Balance ability.” The fourth search field concentrated on methods and techniques for assessing balance function [e.g., the Berg Balance Scale (BBS), the Timed Up and Go test, and the Balance Assessment System test], commonly employed to evaluate the body’s balance capability, with the BBS being notably user-friendly and swift.

**TABLE 1:** Key search words and synonyms used for each search field.

Population	Exercise	Balance function	Measurement technique
TITLE-ABS-KEY	TITLE-ABS-KEY	TITLE-ABS-KEY	TITLE-ABS-KEY
Older adult	Tai Ji	Balance	Berg Balance Scale
Older elderly	Tai chi	Balance function	Timed Up and Go test
Older	Chi, Tai	Balance ability	Balance Evaluation Systems Test
Parkinson’s disease	Yoga	Balance	UPDRS-III
Parkinsonism	Resistance training		
Primary parkinsonism	Strength training		

**FIGURE 1:**
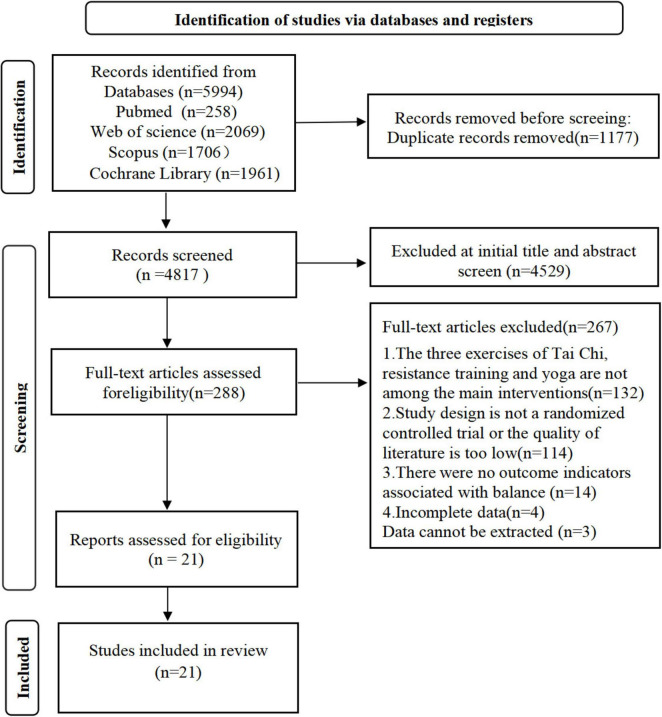
PRISMA flow chart with information at different stages of the study process.

Building on this, we excluded PRT from our review. Although PRT shows a clear correlation with improvements in balance and motor function, we chose to focus primarily on progressive resistance exercise (PRE), yoga, and Tai Chi. These interventions have demonstrated significant effects in the existing literature and are widely applied in clinical practice. All primary search terms were paired with medical subject headings (MeSH) and thoroughly explored. Furthermore, each reference from the included studies underwent scrutiny to ensure the comprehensive coverage of relevant literature. Figure 1 depicts the PRISMA diagram, outlining the literature search strategy and detailing each stage of the research process.

### 2.2 Eligibility criteria

1. Two reviewers (XG and LT) developed the following inclusion and exclusion criteria.
2. The study population comprised healthy older adults aged 50 years and older, as well as patients with mild to moderate PD.
3. In the experimental group, the intervention consisted of various types of exercise interventions or a single type of Tai Chi, resistance training, or yoga. The control group received no exercise intervention, or alternative forms of movement and exercise were utilized.
4. The study incorporated at least one or more of the following assessments: the BBS, Timed Up and Go (TUG) test, movement speed, force plate measurements, and functional balance test.
5. The aim was to investigate the effects of different exercise types (Tai Chi, yoga, and resistance training) on balance function in both healthy older adults and Parkinson’s patients.
6. The outcome indicators were complete and available in full text, and only randomized controlled trials (RCTs) published in the English language were included.
7. Two reviewers, XG and LT, independently assessed the studies based on the above inclusion/exclusion criteria. Initial screening of article titles and abstracts was conducted using NoteExpress 3.9.0.9640 software, with duplicate entries removed. Subsequently, a third reviewer (XYZ) was invited to collaborate in the evaluation of included and uncertain studies.

### 2.3 Quality assessment of studies

**FIGURE 2:**
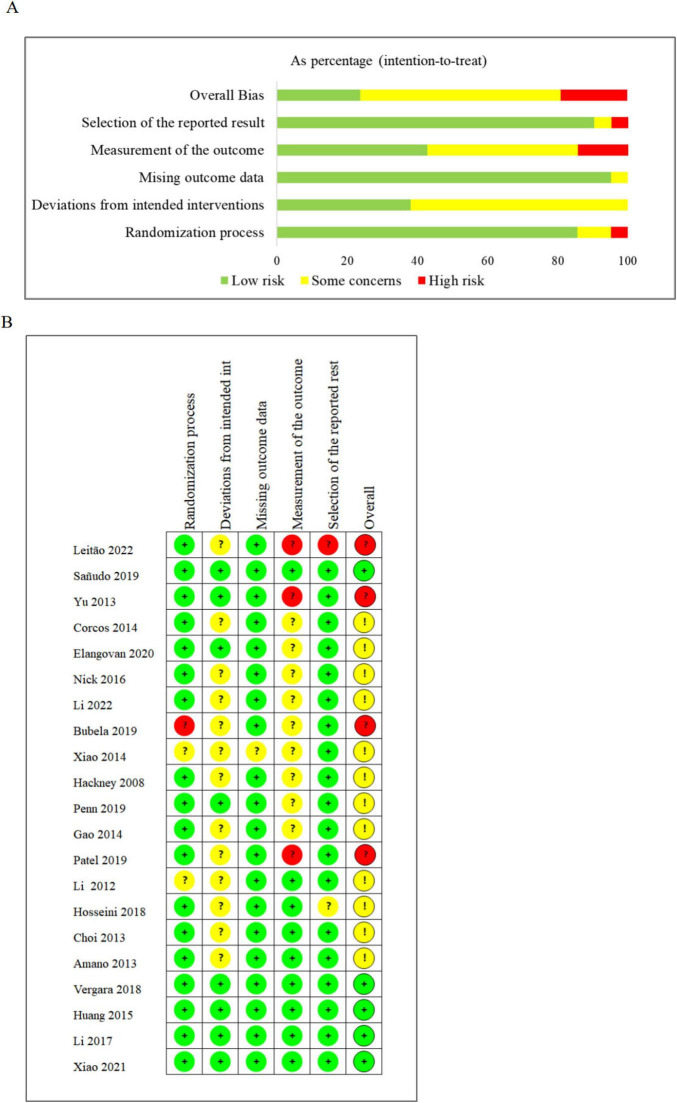
**(A)** Risk of bias graph. **(B)** Risk of bias summary.

Two researchers (XG and LT) independently evaluated the risk of bias and study quality using the Cochrane ROB 2.0 risk of bias tool. As depicted in Figure 2. Parameters assessed encompassed random sequence generation (for randomized grouping), allocation concealment (ensuring confidentiality of randomization sequences), double blinding (for participant and researcher blinding), data completeness (addressing missing data issues), and selective reporting (reporting results comprehensively and avoiding the use of multiple evaluation criteria). Any disagreements or discrepancies were resolved through consultation with a third researcher (XYZ).

### 2.4 Data extraction

Two researchers (XG and LT) independently extracted and synthesized information from articles and data, ensuring objectivity and minimizing bias. A data extraction table was then created, which followed the systematic review of characteristics of similar research topics. The extracted information encompassed the first author’s name, average age, year of publication, intervention program details (method, duration, and frequency), testing method, and main conclusions. The synthesized information is presented in Table 2.

**TABLE 2:** Characteristics of the included studies.

Study year	Mean age (years)	Means of intervention	Intervention dose/rate of completion	Outcomes/major findings
Leitão et al., 2022	Older adults Ages: 67.2 ± 5.1 Study groups: SET (*n* = 20) PWT (*n* = 19) TRT (*n* = 17) AST (*n* = 20) Control (*n* = 19)	Resistance training	60 min session, 2 sessions per week for 16 weeks 95%	BBS All RT protocols improved PA levels and QoL after 16 weeks of training. For the improvement of balance, QoL and PA, older women can be subjected to PWT, AST and SET, and not be restricted to TRT.
Sañudo et al., 2019	Older adults Ages: 64.4 ± 3.61 Study groups: Intervention (*n* = 17) Control (*n* = 17)	Resistance training	60 min session, 2–3 sessions per week for 6 weeks 100%	TUG Flywheel resistance exercise training improved balance and mobility in older adults as well as muscle power.
Yu et al., 2013	Older adults Ages: 65.5 ± 4.5 Study groups: Intervention (*n* = 12) Control (*n* = 12)	Resistance training	60 min session, 3 sessions per week for 5 weeks 100%	BBS TUG The findings of this study indicate that resistance exercise using the Thera-band is possible to improve the static and dynamic balance of elderly adults.
Corcos et al., 2013	Elderly individuals with Parkinson’s disease Ages: 59.0 ± 4.6 Study groups: Intervention (*N* = 24) Control (*N* = 24)	Resistance training	60 min session, 2 sessions per week for 24 months 83.33%	UPDRS-III Movement speed functional PRE demonstrated a statistically and clinically significant reduction in UPDRSIII scores compared to mFC and is recommended as a useful adjunct therapy to improve parkinsonian motor signs.
Elangovan et al., 2020	Elderly individuals with Parkinson’s disease Ages: 64.2 ± 7.5 Study groups: Intervention (*N* = 10) Control (*N* = 10)	Yoga	60 min session, 2 sessions per week for 12 weeks 100%	UPDRS-III Motor scores gait and postural kinematics This study showed that a 12-week Hatha yoga training can improve static balance in PWP. We found no evidence that it systematically improves gait performance in PWP.
Nick et al., 2016	Older adults Ages: 68 ± 4.87 Study groups: Intervention (*N* = 20) Control (*N* = 19)	Yoga	60 min session, 2 sessions per week for 8 weeks 100%	BBS Yoga is a potential intervention to reduce fear of falling and improve balance in older adults.
Li et al., 2022	Elderly individuals with Parkinson’s disease Ages: 50–80 Study groups: Tai Chi (*n* = 32) Brisk walking (*n* = 31) Control (*n* = 17)	Tai Chi	60 min session, 2 sessions per week 12 month 100%	BBS UPDRS-III TUG Long-term Tai Chi training improves motor function, especially gait and balance, in PD.
Bubela et al., 2019	Older adults Ages: 55–86 Study groups: Intervention (*N* = 16) Control (*N* = 13)	Tai Chi	60 min session, 2–3 sessions per week for 16 weeks 100%	TUG Older adults’ participation in a community-based Tai Chi program may lead to improvement in strength, mobility, and confidence in performing functional tasks.
Xiao et al., 2014	Older adults Ages: 60–70 Study groups: Intervention (*N* = 36) Control (*N* = 36)	Tai Chi	12 months, three times a week, for 1–2 h 100%	TUG Tai Chi intervention was both effective for community-dwelling elderly persons in improving balance performance in relation to falls and might delay the onset of deterioration of motor function.
Hackney et al., 2008	Elderly individuals with Parkinson’s disease Ages: 50–78 Study groups: Intervention (*N* = 13) Control (*N* = 13)	Tai Chi	60 min session, 2 sessions per week for 13 weeks 84.62%	UPDRS-III BBS TUG Tai Chi appears to be an appropriate, safe and effective form of exercise for some individuals with mild-moderately severe PD.
Hosseini et al., 2018	Older adults Ages: 60–80 Study groups: Intervention (*N* = 30) Control (*N* = 30)	Tai Chi	55 min session, 2 sessions per week for 8 weeks 100%	TUG Tai Chi Chuan effectively improves the balance and fear of falling and could be considered as a practical and useful method for fall prevention in community-dwelling older adults.
Penn et al., 2019	Older adults Ages: 60–70 Study groups: (iTC) group (*n* = 20) Control (*n* = 15)	Tai Chi	30 min session, 3 sessions per week for 8 weeks 100%	BBS TUG Personalized Tai-Chi training designed based on an objective measurement and conducted according to graded intensity and complexity benefited practitioners after a short period.
Gao et al., 2014	Elderly individuals with Parkinson’s disease Ages: 60–70 Study groups: Intervention (*N* = 37) Control (*N* = 39)	Tai Chi	60 min session, 3 sessions per week for 12 weeks 100%	BBS TUG UPDRS-III Our findings suggested that Tai Chi exercise could improve the balance and decrease the fall risks in patients with Parkinson’s disease.
Patel et al., 2019	Older adults Ages: 60–75 Study groups: Intervention (*N* = 18) Control (*N* = 20)	Yoga	30–40 min session, 5 sessions per week for 4 weeks 100%	TUG The study conducted concludes that yogasanas are effective in improving balance in elderly individuals at the end of 4 weeks compared to control group.
Li et al., 2012	Elderly individuals with Parkinson’s disease Ages: 59–78 Study groups: Tai Chi (*N* = 65) Resistance (*N* = 65)	Tai Chi Resistance	60 min session, 2 sessions per week for 24 weeks 100%	TUG UPDRS-III In conclusion, Tai Chi appears to be effective as a stand-alone behavioral intervention designed to improve postural stability and functional ability in people with Parkinson’s disease.
Choi et al., 2013	Elderly individuals with Parkinson’s disease Ages: 60.81 ± 7.6 Study groups: Tai Chi (*N* = 11) Control (*N* = 9)	Tai Chi	60 min session, 3 sessions per week for 12 weeks 100%	UPDRS-III This study showed that TTC training had modest positive effects on the functional status of Parkinson’s disease patients.
Amano et al., 2013	Elderly individuals with Parkinson’s disease Ages: 54–68 Study groups: Tai Chi (*N* = 12) Control (*N* = 9)	Tai Chi	60 min session, 2 sessions per week for 16 weeks 100%	UPDRS-III Thus the use of short-term Tai Chi exercise should require further study before being considered a valuable therapeutic intervention for these domains in PD.
Vergara-Diaz et al., 2018	Elderly individuals with Parkinson’s disease Ages: 65.7 ± 3.86 Study groups: Tai Chi (*N* = 12) Control (*N* = 13)	Tai Chi	60 min session, 2 sessions per week for 24 weeks 75%	TUG UPDRS-III Conducting an RCT of TC for PD is feasible, though measures to improve recruitment and adherence rates are needed. DT gait STV is a sensitive and logical outcome for evaluating the combined cognitive-motor effects of TC in PD.
Huang et al., 2015	Elderly individuals with Parkinson’s disease Ages: 72.20 ± 7.27 Study groups: Tai Chi (*N* = 10) Control (*N* = 10)	Tai Chi	50–60 min session, 2–3 sessions per week for 12 weeks 100%	BBS UPDRS-III Studies have shown that Tai Chi has positive effects on improving muscle fitness, balance, and preventing falls in older adults. This study investigates the impact of Tai Chi on balance and motor function in patients with early-stage Parkinson’s disease.
Li et al., 2017	Elderly individuals with Parkinson’s disease Ages: 60–80 Study groups: Tai Chi (*N* = 30) Control (*N* = 30)	Tai Chi	60 min session, 4 sessions per week for 12 weeks 100%	BBS Tai Chi training can improve balance and fall self-efficacy in Parkinson’s disease patients, positively impacting their fear of falling.
Xiao et al., 2021	Elderly individuals with Parkinson’s disease Ages: 62–86 Study groups: Tai Chi (*N* = 20) Control (*N* = 20)	Tai Chi	60 min session, 4 sessions per week for 6 months 100%	BBS Incorporating Tai Chi into the clinical treatment of patients with early-stage Parkinson’s disease is highly beneficial for improving their balance impairments. It also enhances their confidence in maintaining balance during activity, which helps prevent and reduce falls, ultimately improving their daily living abilities. The overall advantages are significant, making it a practice worth promoting and applying.

BBS, Berg Balance Scale; UPDRS, Unified Parkinson’s Disease Rating Scale; TUG, Timed Up and Go test.

### 2.5 Data synthesis and statistical analysis

We used Comprehensive Meta-Analysis software, RevMan 5.4.1, to analyze key outcomes. The balance function (BBS) was the primary outcome, while motor functions [TUG and Unified Parkinson’s Disease Rating Scale Part III (UPDRS-III)] were secondary outcomes. Given the high variability of UPDRS-III scores between studies, we used the standardized mean difference (SMD) for the combined analysis. For the analysis of TUG and BBS results, the mean difference (MD) was used, and statistical analyses were conducted using weighted average differences within a 95% confidence interval. In cases of significant heterogeneity among the studies (*p* < 0.05, *I*^2^ > 50%), sensitivity analyses and subgroup analyses were conducted to ensure the robustness of the results and identify the sources of heterogeneity.

## 3 Results

### 3.1 Study selection

An initial search across four databases (PubMed, Web of Science, Scopus, and Cochrane Library) based on the search strategy and keywords yielded a total of 5,994 potentially relevant articles. We utilized a different screening process consisting of (removal of duplicates, initial review, systematic evaluation, title screening, and abstract reading). Please refer to Figure 1 for a detailed outline of the process we employed, 21 articles were identified that met the criteria. These comprised 9 studies involving the healthy elderly population and 12 studies involving patients with PD. Among the healthy elderly population, there were four studies on Tai Chi (Penn et al., 2019; Hosseini et al., 2018; Bubela et al., 2019; Xiao, 2014), three studies on resistance training (Leitão et al., 2022; Sañudo et al., 2019; Yu et al., 2013), and two studies on yoga (Patel et al., 2019; Nick et al., 2016). In contrast, within the PD patient population, there were 10 studies on Tai Chi (Li et al., 2022; Gao et al., 2014; Li et al., 2012; Hackney and Earhart, 2008; Choi et al., 2013; Amano et al., 2013; Vergara-Diaz et al., 2018; Huang et al., 2015; Li et al., 2017; Xiao et al., 2021), 1 studies on yoga (Elangovan et al., 2020), and 1 study focused solely on resistance training (Corcos et al., 2013). Notably, the study by (Li et al., 2012) analyzed Tai Chi and resistance training in comparison.

### 3.2 Characteristics of included studies

Table 2 presents the main characteristics of the studies, screened using the Hoehn and Yahr scales (Hoehn and Yahr, 2001). A total of 21 studies were included. In the studies involving healthy older adults, interventions using Tai Chi ranged from 2 to 3 times per week for 30–120 min per session over 8 weeks to 12 months, while resistance strength training sessions occurred 2–3 times per week for 60 min each, spanning 5–16 weeks. Yoga training sessions varied from 30 to 60 min, occurring 2–5 times per week, lasting from 4 to 8 weeks. For Parkinson’s patients, Tai Chi interventions lasted 60 min, 2–4 times per week, lasting from 8 weeks to 12 months; yoga interventions involved sessions lasting 60 min, twice weekly for 12 weeks, and resistance training sessions lasted 60 min, twice weekly, lasting for 24 weeks.

Among the 21 studies, 10 utilized the BBS as their outcome measure, reflecting balance function (Leitão et al., 2022; Yu et al., 2013; Nick et al., 2016; Penn et al., 2019; Gao et al., 2014; Hackney and Earhart, 2008; Li et al., 2022; Li et al., 2017; Xiao et al., 2021; Huang et al., 2015), including 6 involving Parkinson’s patients (Li et al., 2022; Hackney and Earhart, 2008; Gao et al., 2014; Li et al., 2017; Xiao et al., 2021; Huang et al., 2015). Twelve studies employed the TUG test (Sañudo et al., 2019; Yu et al., 2013; Patel et al., 2019; Bubela et al., 2019; Xiao, 2014; Hosseini et al., 2018; Penn et al., 2019; Li et al., 2022; Gao et al., 2014; Hackney and Earhart, 2008; Li et al., 2012; Vergara-Diaz et al., 2018), with five including Parkinson’s patients (Li et al., 2022; Gao et al., 2014; Li et al., 2012; Hackney and Earhart, 2008; Vergara-Diaz et al., 2018). Furthermore, outcome indicators for both healthy older adults and Parkinson’s patients included the UPDRS, Modified Falls Efficacy Scale (MFES), Fifty-Foot Walk Test (FFWT), and Activities-Specific Balance Confidence (ABC). Researchers primarily utilized UPDRS-III, BBS, TUG to assess participants’ balance and locomotor ability, with UPDRS-III being the predominant method in clinical and research settings (Siderowf et al., 2002).

### 3.3 Methodological quality

Two researchers (XG and LT) performed a literature quality assessment using the Cochrane ROB 2.0 risk of bias tool. The details of each assessment are illustrated in Figure 2. Among the 21 studies reviewed, those exhibiting a higher risk primarily stemmed from the necessity to recruit and group participants from specific sports areas, as evidenced by studies conducted by Yu et al. (2013), Leitão et al. (2022), Bubela et al. (2019), and Patel et al. (2019). Additionally, the implementation of various sports interventions inherently led to participant awareness of intervention content, contributing to some level of risk. However, studies with comparatively lower impact on outcomes were conducted by Nick et al. (2016), Elangovan et al. (2020), Corcos et al. (2013), Sañudo et al. (2019), Xiao (2014), Penn et al. (2019), as well as by Hackney and Earhart (2008), Hosseini et al. (2018), Li et al. (2012), Huang et al. (2015), Li et al. (2017), Xiao et al. (2021), and Vergara-Diaz et al. (2018).

### 3.4 Key findings of healthy older adults

#### 3.4.1 The effect of exercise on balance function

**FIGURE 3:**
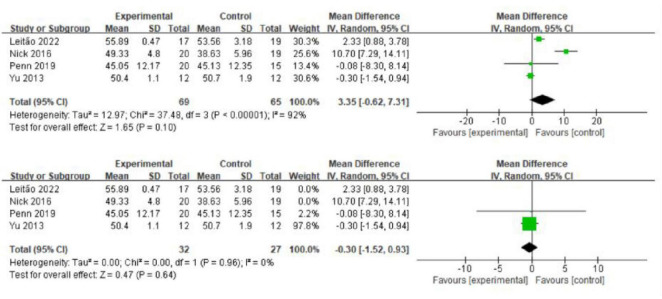
The effect of exercise on balance function.

Four studies with healthy elderly participants used the BBS to evaluate the effects of exercise on balance (Leitão et al., 2022; Yu et al., 2013; Nick et al., 2016; Penn et al., 2019). Despite the significant heterogeneity, the analysis indicated no significant differences in static balance between the intervention and control groups (MD = 3.35; 95% CI = 0.62, 7.31; *p* = 0.10; *I*^2^ = 92%; Figure 3). Removing the (Nick et al., 2016; Leitão et al., 2022), study from a sensitivity analysis eliminated heterogeneity (*I*^2^ = 0%) and provided consistent results (MD = 0.30; 95% CI = 1.52, 0.93; *p* = 0.64; *I*^2^ = 0%; Figure 3).

#### 3.4.2 The effect of exercise on functional walking

**FIGURE 4:**
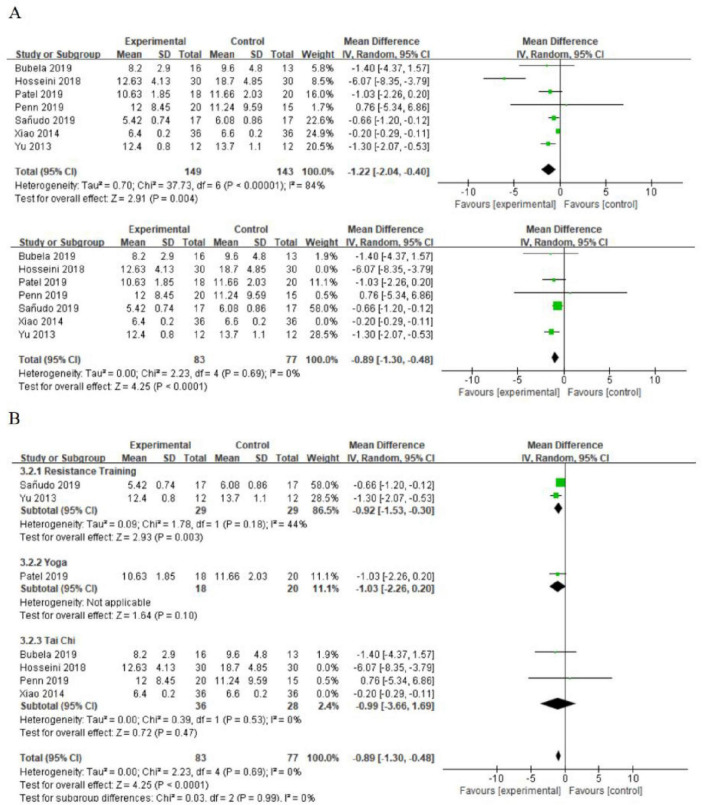
**(A)** The effect of exercise on functional walking. **(B)** The effect of different types of exercise on functional walking (subgroup analysis).

Seven studies utilized the TUG test to evaluate functional walking in older adults (Sañudo et al., 2019; Yu et al., 2013; Patel et al., 2019; Bubela et al., 2019; Xiao, 2014; Hosseini et al., 2018; Penn et al., 2019). A high degree of heterogeneity was identified, with an *I*^2^ value of 84%. Upon investigation, studies by Xiao (2014) and Hosseini (2018) were identified as sources of heterogeneity. Excluding these studies significantly decreased the heterogeneity to *I*^2^ = 0%. Compared to the control group, the three types of exercise significantly improved functional walking (MD = 0.89; 95% CI = 1.30, 0.48; *p* < 0.0001; *I*^2^ = 0%; Figure 4). Further subgroup analysis revealed statistically significant differences in TUG test scores across different exercise interventions. Resistance training showed a more favorable effect than the control group (MD = 0.92; 95% CI = 1.53, 0.30; *p* = 0.003). However, yoga and Tai Chi did not show statistically significant differences in TUG test scores compared to the control group (yoga: MD = 1.03; 95% CI = 2.26, 0.20; *p* = 0.10) and (Tai Chi: MD = 0.99; 95% CI = 3.66, 1.69; *p* = 0.47; Figure 4).

In summary, although all three types of exercise interventions significantly improved balance function in older adults, the BBS balance scale showed no significant changes in healthy older adults. Meta-analysis using the TUG measure revealed significant improvements in balance, with resistance training being particularly effective.

### 3.5 Key findings of people with PD

#### 3.5.1 The effect of exercise on motor function

**FIGURE 5:**
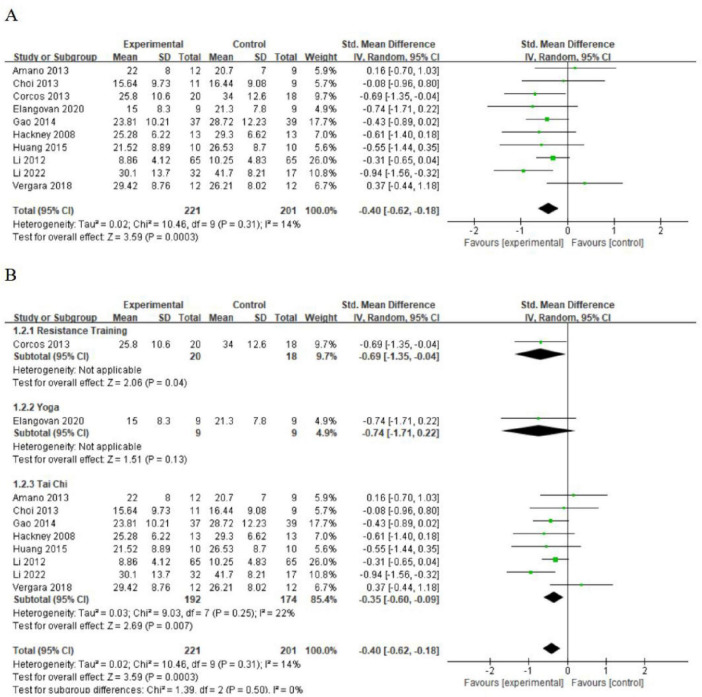
**(A)** The effect of exercise on motor function. **(B)** The effect of different types of exercise on motor function (subgroup analysis).

In the PD group, 10 studies utilized the UPDRS-III to assess motor function (Corcos et al., 2013; Elangovan et al., 2020; Li et al., 2012; Gao et al., 2014; Hackney and Earhart, 2008; Li et al., 2022; Choi et al., 2013; Amano et al., 2013; Vergara-Diaz et al., 2018; Huang et al., 2015). Results indicated that all three types of exercise improved functional walking compared to the control group (SMD = 0.40; 95% CI = 0.62, 0.18; *p* = 0.0003; *I*^2^ = 14%; Figure 5). Given the different exercise interventions, we conducted subgroup analyses. The meta-analysis revealed varying effectiveness among the exercise types in improving functional walking. Resistance training (SMD = 0.69; 95% CI = 1.35, 0.04; *p* = 0.04) and Tai Chi (SMD = 0.35; 95% CI = 0.60, 0.09; *p* = 0.007) had positive effects. However, yoga did not show a statistically significant difference from the control group (SMD = 0.74; 95% CI = 1.71, 0.22; *p* = 0.13; Figure 5).

#### 3.5.2 The effect of exercise on balance function

**FIGURE 6:**
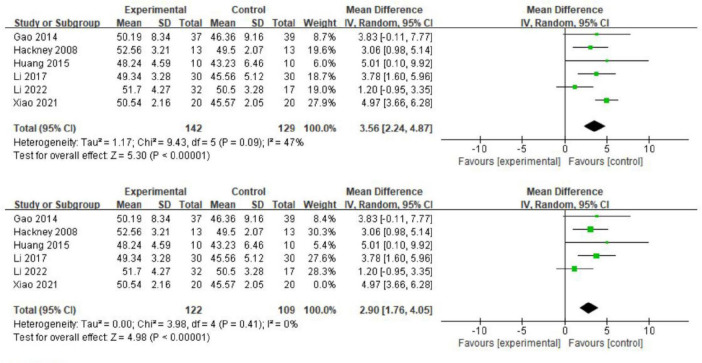
The effect of exercise on balance function.

Six studies have employed the BBS to assess balance function in patients with PD (Gao et al., 2014; Li et al., 2022; Hackney and Earhart, 2008; Li et al., 2017; Xiao et al., 2021; Huang et al., 2015). A meta-analysis revealed significant heterogeneity (*I*^2^ = 47%) and showed that Tai Chi significantly improved balance function in PD patients compared to the control group (MD = 3.56; 95% CI = 2.24, 4.87; *p* < 0.00001; Figure 6). Sensitivity analysis, excluding the study by Xiao (2021), resulted in reduced heterogeneity (*I*^2^ = 0%) while maintaining the same results (MD = 2.90; 95% CI = 1.76, 4.05; *p* < 0.00001; Figure 6).

**FIGURE 7:**
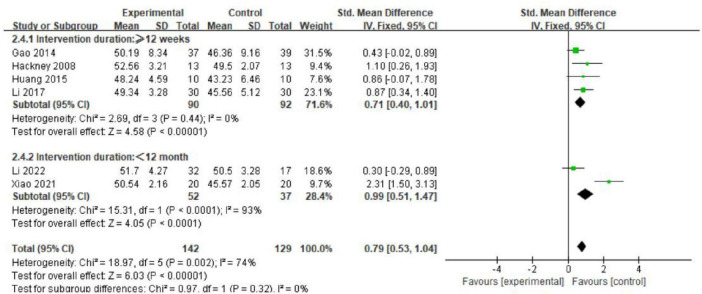
The effects of different intervention durations on balance function (subgroup analysis).

**FIGURE 8:**
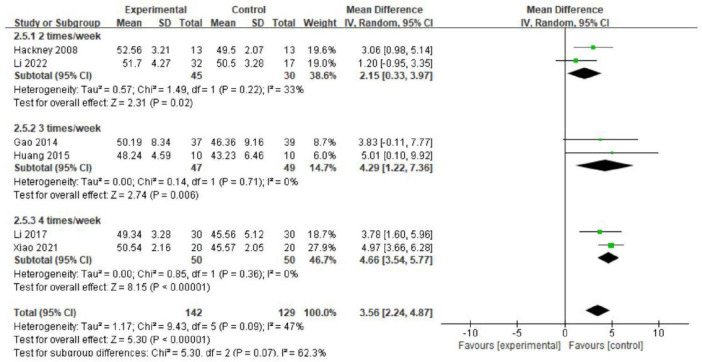
The effects of different intervention frequencies on balance function (subgroup analysis).

We conducted further subgroup analysis to assess the effects of Tai Chi intervention duration and frequency on balance function in PD patients. The results indicated no significant differences among various intervention durations (MD = 0.79; 95% CI = 0.53, 1.04; *p* = 0.32, *I*^2^ = 0%; Figure 7). Analysis of intervention dosage and exercise frequency showed that engaging in Tai Chi four times per week was most effective (MD = 4.66; 95% CI = 3.54, 5.77; *p* < 0.00001, *I*^2^ = 0%; Figure 8). Conversely, interventions conducted twice and three times per week had *p* values of 0.02 and 0.006.

#### 3.5.3 The effect of exercise on functional walking

**FIGURE 9:**
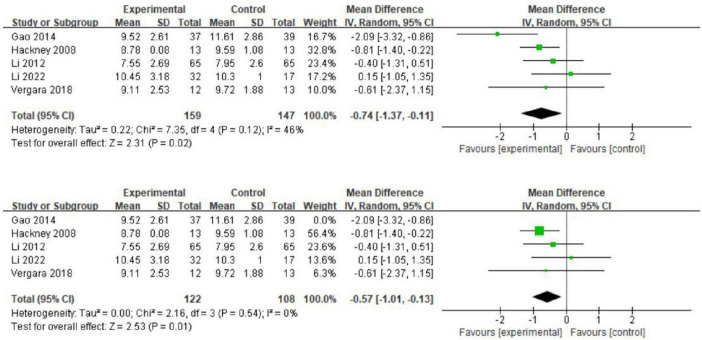
The effect of exercise on functional walking.

In the group of patients with PD, five studies used the TUG test to assess patients’ functional walking (Li et al., 2022; Gao et al., 2014; Hackney and Earhart, 2008; Li et al., 2012; Vergara-Diaz et al., 2018). Compared to the control group, Tai Chi exercises improved functional walking (MD = 0.74; 95% CI = 1.37, 0.11; *p* = 0.02; *I*^2^ = 46%; Figure 9). Sensitivity analysis showed that heterogeneity would significantly decrease only by excluding the study by Gao (2014), but the results remained unchanged (MD = 0.57; 95% CI = 1.01, 0.13; *p* = 0.01; *I*^2^ = 0%). This finding indicates that the study by Gao (2014), was a major contributor to the high heterogeneity.

Overall, these 12 studies evaluated the effects of three types of exercise interventions on patients with PD. First, a meta-analysis assessing balance using the TUG test demonstrated significant improvement in the Tai Chi intervention group compared to the control group. Subgroup analysis by intervention frequency indicated that this improvement did not increase significantly over time. Additionally, a subgroup analysis of the UPDRS-III showed that Tai Chi and resistance training significantly enhanced motor function.

## 4 Discussion

**FIGURE 10:**
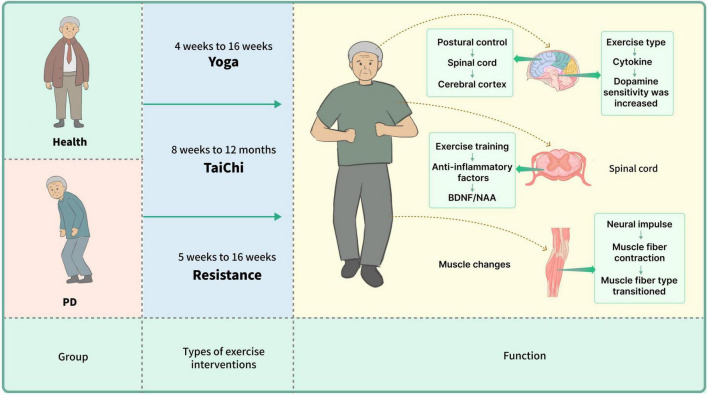
The balance function of healthy elderly individuals and patients with Parkinson’s disease significantly improved after sustained yoga practice for 4–16 weeks, Tai Chi practice for 8–12 months, or resistance training for 5–16 weeks. During these training processes, changes in body posture positively impacted the spinal cord and cerebral cortex; exercise training led to an increase in anti-inflammatory factors, which further influenced the increase in BDNF/NAA and other cytokines, thereby enhancing dopamine plasticity. Long-term practice also resulted in a transformation of muscle fiber contraction types.

This systematic review and meta-analysis indicate that all three types of exercise interventions can significantly enhance balance and motor functions in older adults. In healthy older adults, resistance training shows the most substantial effect in improving dynamic balance. For PD patients, both resistance training and Tai Chi significantly enhance motor function and balance. Continuous Tai Chi intervention significantly improves the balance of PD patients. The effects become increasingly significant with higher intervention frequency (*p* < 0.00001). While there is no significant change in effect over time (*p* = 0.32), interventions conducted 3–4 times per week, for 50–60 min each session, over 12 weeks, yield the most significant improvements in balance. Furthermore, as illustrated in Figure 10, our review synthesized these exercise interventions’ behavioral, functional, and mechanical benefits for healthy older adults and PD patients.

In nine studies with healthy older adults, Tai Chi, yoga, and resistance exercise significantly improved gait, flexibility, stability, and tissue-level functioning. Improvements in dopamine levels were also noted.

The BBS, TUG test, and gait measures are commonly used to evaluate balance in Tai Chi studies. These tools have shown significant improvements in lower limb muscles, flexibility, and functional tasks, indicating that Tai Chi may slow motor function decline. These studies provide strong evidence to address key concerns (Hosseini et al., 2018). Tai Chi improves proprioception in the trunk and lower limbs and enhances balance by integrating vestibular and visual inputs (Bubela et al., 2019; Xiao, 2014). Personalized Tai Chi interventions have shown the highest efficacy (Penn et al., 2019). In the yoga intervention group, two studies found that practitioners performed rhythmic stretches with breath control, joint mobilization, and muscle strengthening. Yoga techniques, including posture adjustments and breathing exercises, effectively enhance strength, flexibility, and balance by improving posture control and proprioception (Patel et al., 2019; Nick et al., 2016). Strength training studies assessed overload progression by evaluating maximal repetitions of static balancing and swaying amplitude. Two studies highlighted that different resistance programs varied in effectiveness, with strength training particularly improving daily mobility and quality of life. Leitão et al. (2022) and Yu et al. (2013) found that resistance exercises improved balance, quality of life, and mobility in older adults. Sañudo et al. (2019) also reported improvements in gait, mobility, and static balance, suggesting that resistance training enhances balance, mobility, and muscle strength.

Twelve studies on Parkinson’s patients explored Tai Chi, yoga, and resistance exercise. These interventions showed improvements in behavioral symptoms (e.g., dystonia and motor difficulties), motor functions (e.g., gait and balance), and overall quality of life.

Two studies on Tai Chi showed initial effects were minor, but notable improvements in bradykinesia, muscle tone, posture, and functional activity were observed later, indicating that extended practice may be necessary for significant benefits (Vergara-Diaz et al., 2018; Choi et al., 2013). Li et al. (2012) found Tai Chi outperformed stretching and resistance training in stride length and functional stretching. (Gao et al., 2014) also showed Tai Chi improved gait, postural regulation, body strength, and posture compared to controls. This improvement was linked to Tai Chi’s combination of fluidity and stillness, which enhanced body strength and range of motion (Hackney and Earhart, 2008). Enhanced balance, visual network function, and metrics such as the BBS and reduced interleukin-1 beta levels were observed (Huang et al., 2015; Li et al., 2017). Improvements in UPDRS scores were also related to better visual network function. Tai Chi training significantly improved metabolic pathways, including arginine biosynthesis, the urea cycle, the tricarboxylic acid cycle, and β-oxidation of ultra-long-chain fatty acids (Li et al., 2022). Intensive resistance training significantly improved strength, motor agility, physical function, and quality of life in Parkinson’s patients. Well-structured long-term exercise programs have demonstrated substantial benefits for motor function (Corcos et al., 2013). Yoga interventions, evaluated for gait, postural fluency, UPDRS, cadence, walking speed, and turnaround time, showed improvements in static balance but minimal changes in gait (Elangovan et al., 2020). Corcos et al. (2013) found prolonged exercise effective in reducing Parkinson’s symptoms, while Elangovan et al. (2020) reported that Hatha yoga improved static balance but had little effect on gait. The variability in results across studies, including inconsistent changes in gait (Myers et al., 2020; Patel et al., 2019; Nick et al., 2016; Bartos et al., 2022), maybe due to differences in assessment methods and intervention protocols. Differences in study design, such as intervention duration and frequency, contribute to these discrepancies. Myers et al. (2020) focused on PD patients, whereas other studies targeted general older adults. For instance, Bartos et al. (2022) used a 16-week program with bi-weekly sessions, Patel et al. (2019) a 4-week program with five sessions per week, and Elangovan et al. (2020) a 12-week program with bi-weekly sessions. These variations likely account for the observed differences in outcomes.

Completion rates varied across studies, as shown in Table 2. Most interventions were conducted 2–3 times per week, with durations ranging from a few weeks to 24 months; longer interventions may yield more pronounced effects. Sessions typically lasted around 60 min, though some varied between 30 and 90 min, potentially affecting participant tolerance and outcomes. High completion rates, such as 100% in studies by Sañudo et al. (2019), Yu et al. (2013), and Li et al. (2022), indicate strong participant acceptance and adherence. Conversely, lower completion rates were observed in studies like Leitão et al. (2022), which involved resistance training for healthy older adults (60 min/session, 2 times per week for 16 weeks; 1 of 20 participants dropped out), and Vieira and Cavalcante (2021), which examined Tai Chi for Parkinson’s patients (60 min/session, 2 times per week for 24 weeks; only 12 of 16 participants completed the intervention after 6 months). These lower rates may reflect challenges in intervention implementation or issues with long-term adherence.

It is important to note that the extensive research on Tai Chi and yoga may overshadow the potential benefits of resistance training in improving balance functions. While existing studies suggest that resistance training might have unique effects on PD patients, the limited literature prevents a comprehensive assessment. This imbalance in available research could introduce bias when comparing the overall effects of different physical activities—such as Tai Chi, yoga, and resistance training—on balance function, potentially leading to an overemphasis or underestimation of the effectiveness of Tai Chi or yoga interventions.

Our investigation shows that self-posture changes during movement are primarily controlled by the spinal cord, improving balance post-training (Taube et al., 2007). This regulation involves the cerebral cortex, cerebellum, and brainstem (Takakusaki, 2017; Richer and Lajoie, 2020). Exercise enhances cognitive functions by reducing inflammation, increasing anti-inflammatory cytokines, and boosting BDNF and NAA production, which improve neural plasticity (Tian et al., 2017; Èekanauskaitë et al., 2020). Zhu et al. (2023) linked improved balance to increased vestibular nucleus functionality. PD patients show deviations in the default mode network (DMN) affecting memory regions (Pedersini et al., 2020). Conflicting sensory inputs can impact gait and balance, potentially enhancing overall stability.

Studies by Jarvis (2017), Assländer et al. (2015), and Rogge et al. (2018) indicate that movement increases dopamine and serotonin transporters, altering brain chemistry. Tai Chi training improves brain network function, and neurotransmitter metabolism, and reduces dopaminergic degeneration in PD patients (Su et al., 2018). Additionally, Tai Chi, yoga, and resistance exercises enhance postural control and proprioceptive and visual systems, providing relief from neurodegenerative disorders (Xu, 2004; Peterka, 2002; Koelewijn and Ijspeert, 2020).

Based on this systematic review, Tai Chi, yoga, and resistance exercises have positively impacted balance, visuospatial skills, gait, and stride in both healthy older adults and PD patients.

Despite evidence suggesting that exercise improves motor function and benefits balance, cognitive function, gait, posture control, proprioception, and neurotransmitter metabolism in both patients and healthy individuals, the exact mechanisms remain unclear. Increased brain connectivity is a key theoretical basis for these benefits, but this is supported mainly by indirect evidence. Our review of 21 studies on exercise interventions for balance shows that while Tai Chi, yoga, and resistance exercises can alleviate PD symptoms and enhance motor performance, the generalizability of these findings is limited. Variability in study content, such as differing assessment tools and methods, and the fact that only 12 studies involved PD patients, restrict our comprehensive understanding. Additionally, diverse intervention plans with varying frequency, intensity, duration, and types further complicate the identification of the most effective exercise regimens.

Future research should focus on several key areas to enhance our understanding of exercise interventions. First, standardizing exercise protocols, including frequency, intensity, duration, and type, is crucial for ensuring consistent and comparable assessments across studies. Second, it is important to investigate the underlying mechanisms of exercise benefits using advanced neuroimaging and biomarker analyses to gain clearer insights into the biological effects. Third, accounting for individual variability in physical activities can help tailor intervention strategies to different populations. Finally, addressing knowledge gaps by exploring under-researched areas, such as PRT for PD patients, will contribute to a more comprehensive understanding of exercise effects.

## 5 Conclusion

This systematic review and meta-analysis indicate that Tai Chi, yoga, and resistance training significantly enhance brain network function in both healthy older adults and patients with PD. These exercises improve connectivity in subcortical structures, thus enhancing balance and motor function. The study identified resistance training as having the most significant effect on improving balance in healthy older adults, while Tai Chi proved particularly effective for Parkinson’s patients. It is recommended that these exercises be practiced for 50–60 min, three to four times a week, and continued for at least 12 weeks to achieve optimal results.

## Data Availability

The raw data supporting the conclusions of this article will be made available by the authors, without undue reservation.
